# Headache determines quality of life in idiopathic intracranial hypertension

**DOI:** 10.1186/s10194-015-0521-9

**Published:** 2015-05-15

**Authors:** Yasmeen Mulla, Keira A Markey, Rebecca L Woolley, Smitaa Patel, Susan P Mollan, Alexandra J Sinclair

**Affiliations:** Neurosciences, School of Clinical and Experimental Medicine, College of Medical and Dental Sciences, University of Birmingham, Vincent Drive, Birmingham, B15 2TT UK; Birmingham Clinical Trials Unit (BCTU), School of Health and Population Sciences, University of Birmingham, Birmingham, UK; Birmingham Neuro-Ophthalmology Unit, Ophthalmology Department, University Hospitals Birmingham NHS Trust, Queen Elizabeth Hospital Birmingham, Birmingham, UK

**Keywords:** Idiopathic intracranial hypertension, Headache, Quality of life, SF-36

## Abstract

**Background:**

The effect of idiopathic intracranial hypertension (IIH) on quality of life (QOL) is poorly understood. Our objectives were to compare QOL in IIH to the normal UK population; to investigate QOL changes with treatment of IIH, using a weight loss intervention, and to determine which clinical factors influence QOL.

**Methods:**

This was a prospective cohort evaluation of QOL, using the 36-Item Short Form (SF-36) Health Survey questionnaire, before and after a therapeutic dietary intervention which resulted in significant reduction in body mass index (BMI), intracranial pressure (ICP), papilloedema, visual acuity, perimetric mean deviation (Humphrey 24–2) and headache (six-item headache impact test (HIT-6) and headache diary). Baseline QOL was compared to an age and gender matched population. The relationship between each clinical outcome and change in QOL was evaluated.

**Results:**

At baseline, QOL was significantly lower in IIH compared to an age and gender matched population in most domains, p < 0.001. Therapeutic weight loss led to a significant improvement in 10 out of 11 QOL domains in conjunction with the previously published data demonstrating significant improvement in papilloedema, visual acuity, perimetry and headache (p < 0.001) and large effect size. Despite significant improvement in clinical measures only headache correlated significantly (p < 0.001) with improving QOL domains.

**Conclusions:**

QOL in IIH patients is significantly reduced. It improved with weight loss alongside significant improvement in clinical measures and headache. However, headache was the only clinical outcome that correlated with enhanced QOL. Effective headache management is required to improve QOL in IIH.

**Electronic supplementary material:**

The online version of this article (doi:10.1186/s10194-015-0521-9) contains supplementary material, which is available to authorized users.

## Background

Idiopathic intracranial hypertension (IIH), also known as primary pseudotumour cerebri and previously benign intracranial hypertension, is characterised by increased intracranial pressure (ICP) and papilloedema in the setting of brain imaging with no evidence of space occupying lesion or venous thrombosis [1,2]. The condition commonly affects young, obese women (incidence in obese female population 11-21/100,000/year) and causes headaches and visual loss [3-5]. To date clinical and research outcomes have focussed on vision and to a lesser extent headache. The previously reported rates of visual loss (25% permanent severe blindness) are likely to be an overestimate, particularly in specialist centres: yet many IIH patients remain very disabled by the condition [6,7].

There is a paucity of the literature evaluating QOL in IIH with currently only three previous published studies [8-10]. QOL in IIH is not different compared to an age, gender and BMI matched population in the domains of vitality, mental health and role limitation due to emotional problems, implying there are factors besides obesity in determining QOL in IIH [8]. QOL has been shown to improve following treatment with acetazolamide, however it was unclear which factors contributed to the improvement in QOL [10]. QOL in IIH has also been shown to be impaired compared to other neuro-ophthalmic disorders [9].

Our objective was to characterise QOL in an adult IIH cohort in the United Kingdom and compare it to an age and gender matched normal population. We then sought to assess QOL in the cohort following a therapeutic dietary intervention. QOL parameters were compared to clinical outcome measures to evaluate which factors were associated with positive changes in QOL.

## Methods

Evaluations were conducted alongside a previously reported prospective multicentre cohort study which documents the full protocol [11]. In summary, twenty five females with IIH, over the age of 16 years, consented to participate in the study. IIH was diagnosed in accordance with the updated modified Dandy Criteria [2]. All participants had a disease duration of 3 months and signs of active disease with raised ICP pressure >25 cmCSF and papilloedema. Magnetic resonance imaging and venography did not reveal alternative pathology in all those recruited.

### Study design

The study was a prospective cohort evaluation consisting of two stages each lasting 3 months with a follow up visit at 9 months: Stage 1 (0–3 months): no intervention and Stage 2 (3–6 months): low calorie diet. The intervention period involved each woman receiving a nutritionally complete low calorie liquid replacement diet (Lipotrim, Howard Foundation, Cambridge) of 425 kcal/day.

Subjects were evaluated at baseline, 3 months, 6 months and a follow up visit at 9 months. The primary outcome measure was quality of life, using the validated 36-Item Short Form Health Survey questionnaire (version 1) [12-14]. This survey consists of 36 questions which evaluate eight dimensions of health: physical functioning, role limitation due to physical problems, role limitation due to emotional problems, social functioning, mental health, energy/vitality, pain and general health perception [12]. A physical component score and a mental component score can be calculated in order to summarise the health dimensions [14]. There is a further question asking patients about their change in health (not included for normative data). The scoring scale is from 0 (worst QOL) to 100 (best QOL) [14] Other clinical measures were evaluated as previously described [11]. Assessment of visual function was carried out using a LogMAR (log of the minimum angle of resolution) chart to assess visual acuity and automated perimetry (Humphrey 24–2 central threshold) to measure the visual field mean deviation. Papilloedema was evaluated using ocular coherence tomography (OCT) (Stratus OCTTM V4.0.1, Carl Zeiss, Meditec, Welwyn Garden City, UK), average retinal nerve fibre layer thickness. A daily headache diary, completed in the week prior to each visit, evaluated headache severity using a visual analogue pain scale (VAS), headache frequency (days/week) and use of analgesia (days/week). A headache questionnaire (Headache Impact test (HIT-6) score range 36–78) was also completed. ICP was measured by lumbar puncture by the same physician (AJS) at baseline, 3 and 6 months after all listed outcome measures had been assessed. Measurements were taken with the patient breathing steadily in the left lateral position, legs extended greater than 90° at the hip, allowing adequate time to ensure a stable ICP reading.

### Data and statistical analysis

The statistical analyses were performed using the statistical package SAS version 9.2 (Cary, NC, USA), Stata version 13 (Statacorp, Texas, USA) and GraphPad Prism version 5. Missing data were excluded from analyses. We used two-tailed p values with p < 0.05 considered statistically significant. We have not adjusted for multiple testing due to the fact that the SF-36 domains are not independent of one another, indeed the component scores are calculated based on the domain scores. This reduces the probability of us finding a significant domain by chance. We have, however, additionally stated in the tables the threshold p values following a Bonferroni correction to adjust for the multiple tests for the readers interest. For longitudinal evaluation, the magnitude of change from baseline to the end of the intervention was addressed with a measure of effect size. Comparison SF-36 data was obtained from published normative data in the United Kingdom (The Oxford Health and Lifestyles Survey 1991–1992) [15]. The data was matched for gender and we used a linear regression model, adjusting for age, to compare the normal population data with the trial data. QOL changes with therapeutic diet were compared at baseline and 3 months; and 3 months and 6 months: these were analysed using paired t-tests. We correlated the changes in QOL with clinical measures and obtained Pearson’s coefficient to ascertain a linear relationship.

### Statement of ethical approval and consent

This study was approved by the Dudley local research ethics committee [06/Q2702/64] and informed written consent was given by all participants.

## Results

**Figure 1:**
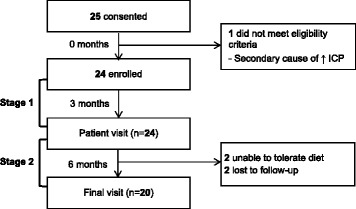
Study design flowchart.

25 women entered the study and 20 completed the study. Figure 1 details numbers of individuals and reasons for non-participation at each stage of the study. Our study included females only and had an age range of 19–54 years Baseline characteristics are shown in Table 1.

**Table 1:** **Baseline demographics**

	**Mean (SD)**
**Age in years**	34.4 (9.2)
**Duration of disease in months**	39.0 (49.2)
**Weight (kg)**	101.5 (16.0)
**Body Mass Index (kg/m2)**	38.2 (5.0)
**Intracranial pressure (cm CSF)**	39.8 (5.1)
	**Number (%)**
**Taking acetazolamide**	11 (44)
**Ethnicity**	
-White	20 (80)
-African-Caribbean	3 (12)
-South West Asian (Indo-Pakistani)	2 (8)

### Quality of life in idiopathic intracranial hypertension

**Figure 2:**
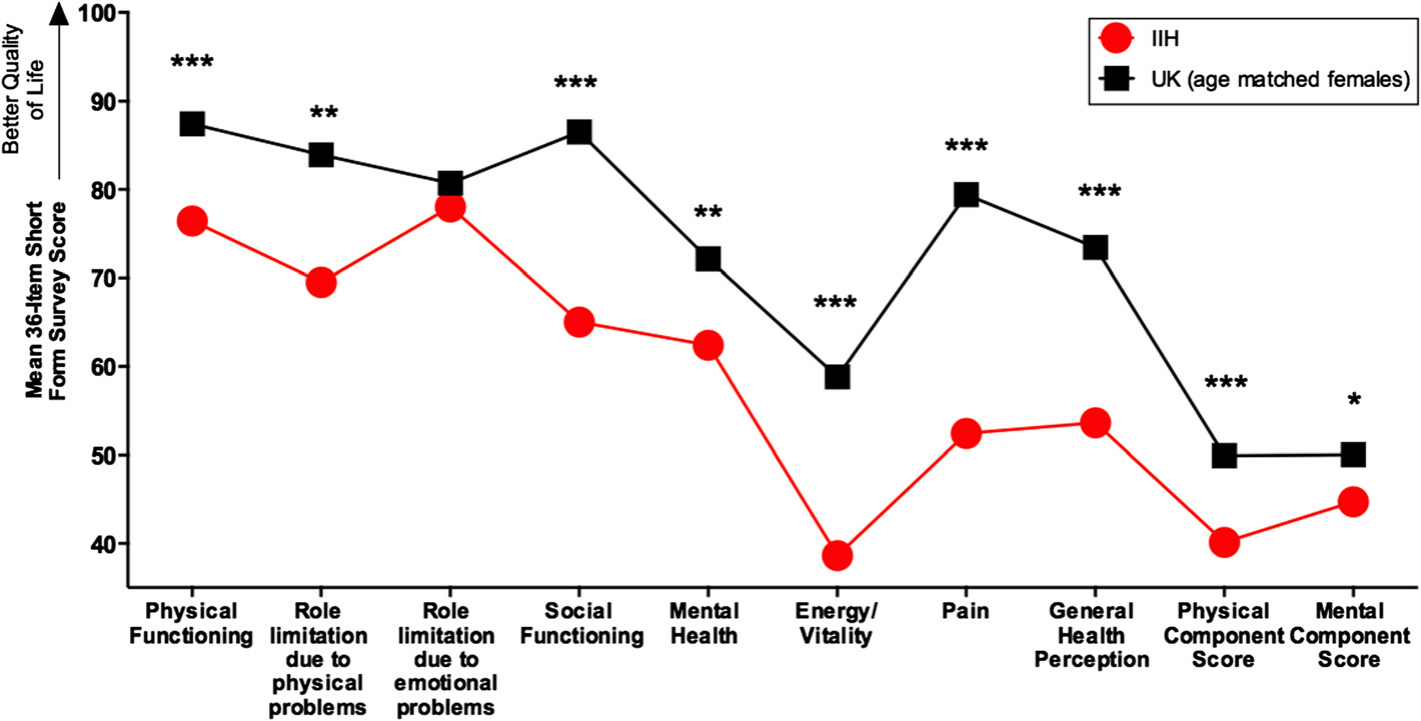
Mean short-form survey scores for IIH (baseline) and UK age matched females. P-values for the difference in means are show, *indicates p < 0.05, **indicates p < 0.01 and ***indicates p < 0.001.

At baseline, mean 36-Item Short Form Survey scores were significantly lower in IIH patients (n=24) in 9 of the 10 domains compared to normative data (a gender and age matched UK population, n=3338) (Figure 2). The IIH SF-36 summary scores were compared. The mean difference of IIH versus a matched age and gender UK population for physical component score was 10.6 (95% CI: 6.7 to 14.5), p < 0.001, but the mean difference for IIH versus a matched age and gender UK population for mental component score was not significant. 4.8 (95% CI: 0.3 to 9.3), p=0.036.

### Quality of life changes with therapeutic diet

The mean differences in scores were evaluated from the beginning to the end of each stage. Our previously published data demonstrates that there was no significant improvement over the observation period (stage 1) but following the diet (stage 2) there was significant improvement in weight, BMI, ICP, papilloedema, visual acuity, headache disability (HIT-6) as well as headache severity and frequency (Table 2). Analgesic use also significantly reduced (p=0.007). Weight, BMI, ICP and HIT-6 showed a particularly large magnitude of change, with effect sizes greater than 0.80.

**Table 2:** **Clinical Outcomes and Quality of Life means (SD) at 0 months (baseline), 3 months and 6 months in Idiopathic intracranial hypertensi**on

	**0 months (baseline)**	**3 months**	**6 months**		
	**Mean (SD)**	**Mean (SD)**	**Mean (SD)**	**P-value**	**Effect size**
**Clinical outcomes**					
Weight (kg)	101.5 (16.0)	102.5 (16.8)	86.8 (15.6)	<0.001	0.92
BMI	38.2 (5.0)	38.6 (5.3)	32.6 (4.7)	<0.001	1.12
Intracranial pressure (cm H_2_O)	39.8 (5.1)	38.0 (5.0)	30.0 (4.9)	<0.001	1.92
Headache disability (HIT-6)	57.5 (9.0)	54.5 (1.0)	46.9 (10.1)	0.004	1.18
Headache severity (VAS)	3.8 (2.4)	4.2 (2.8)	1.9 (2.8)	0.015	0.79
Headache frequency (days/week)	3.8 (2.9)	4.4 (2.9)	2.1 (2.8)	0.011	0.59
Papilloedema (OCT)	144.1 (45.5)	135.0 (48.0)	109.3 (27.9)	0.001	0.76
Visual acuity (LogMar)	-0.02 (0.10)	0.01 (0.11)	-0.06 (0.09)	<0.001	0.40
Analgesic use (days/week)	2.2 (2.7)	2.2 (2.5)	0.2 (0.4)	0.007	0.74
**Quality of life (SF-36)**					
Physical functioning	77.3 (22.9)	79.8 (21.2)	87.6 (19.4)	<0.001	-0.45
Role limitation due to physical problems	70.6 (29.3)	78.1 (24.7)	91.4 (16.9)	<0.001	-0.71
Role limitation due to emotional problems	78.8 (30.0)	76.0 (28.8)	90.1 (18.7)	0.018	-0.38
Social functioning	66.7 (30.3)	78.1 (19.6)	86.9 (22.8)	0.081	-0.67
Mental health	63.2 (22.4)	67.2 (20.5)	74.6 (21.3)	0.048	-0.51
Energy/Vitality	39.4 (20.8)	49.6 (14.4)	71.0 (23.4)	<0.001	-1.52
Pain	53.2 (20.2)	67.6 (26.8)	85.2 (16.2)	0.001	-1.58
General health perception	53.1 (18.8)	53.7 (18.6)	72.1 (12.7)	0.001	-1.01
Change in health	52.1 (19.4)	60.4 (19.4)	88.1 (17.0)	<0.001	-1.86
Physical component score	40.4 (10.3)	44.1 (10.5)	50.7 (7.4)	<0.001	-1.00
Mental component score	45.2 (12.7)	47.1 (9.8)	52.(10.4)	0.020	-0.54
Number	24	24	20		

HIT-6, Six-item Headache Impact Test; OCT, Optical Coherence Tomography. VAS, Visual Analogue Score; LogMar, Logarithm of the Minimum Angle of Resolution. The quality of life scale ranges from 0 (worst quality of life) to 100 (best quality of life). There were no significant differences between 0 and 3 months (no diet), except for pain (p=0.025) and energy/vitality (p=0.020). P values in the table indicate changes from 3 to 6 months (following diet). If a Bonferroni correction were applied the p value is reduced to p < 0.0026, but this might represent an overcorrection. Effect size (ES) in the final column indicates the magnitude of change: ES=0.30 indicates mild/low change, ES=0.50 indicates moderate change and ES ≥ 0.80 indicates large change.

The 36-Item Short Form Health Survey domains did not improve significantly during stage 1 (no intervention), except for pain (mean change 14.4, p=0.025) and energy/vitality (mean change 10.2, p=0.020). Following therapeutic weight loss (stage 2, low energy diet) the mean differences in 36-Item Short Form Health Survey scores during stage 2 improved significantly in 10 of 11 domains (Additional file 1). The magnitude of change was particularly large (ES ≥ 0.80) for 5 out of the 11 domains: energy/vitality, pain, general health perception, change in health and physical component score.

We found that there were no significant changes in quality of life 3 months after the dietary intervention had finished, suggesting that the improvement in quality of life was sustained in this period. The change in SF-36 domains do not differ between those who use and do not use acetazolamide (Diamox) for any of the domains.

### Relationship between quality of life and clinical outcomes

The linear relationship between the changes in clinical outcomes measures and changes 36-Item Short Form Health Survey domains following the dietary intervention, were then assessed. Despite the significant improvement in visual function, reduction in ICP, papilloedema, BMI and reduction in weight during the dietary intervention, none of these measures correlated with the improvement in QOL scores (Additional file 2).

Interestingly the 36-Item Short Form Health Survey correlated strongly with an improvement in headaches outcomes: Headache Impact Test scores (HIT-6) correlated significantly with all domains of the 36-Item Short Form Health Survey (Additional file 3). In particular, the mental health component score, summarising mental health dimensions (r=-0.88, p < 0.001), the social functioning domain (r=-0.81, p < 0.001) and the energy/vitality domain (r=-0.79, p < 0.001). Headache severity correlated significantly with 10 out of 11 domains, particularly role limitation due to emotional problems (r=-0.78, p < 0.001), mental component score role (r=-0.73, p=0.002) and limitation due to physical problems (r=-0.71, p=0.002) (Table 3). Headache frequency correlated with an improvement in Short Form Health Survey scores which was significant for only 5 out of the 11 domains (p < 0.05) and the correlation was weaker than headache disability (HIT-6) and headache severity (Table 3).

**Table 3:** **Correlation between changes in the 36-item short form health survey domains and changes in headache disability, severity and frequency following the therapeutic diet**

**Domain**	**Headache disability (Headache impact test-6 score)**	**Headache severity (Visual analogue pain scale)**	**Headache frequency (days/week)**
**Physical functioning**	-0.67	-0.74	-0.48
	p=0.001	p=0.001	p=0.052
**Role limitation due to physical problems**	-0.70	-0.71	-0.53
	p < 0.001	p=0.002	p=0.029
**Role limitation due to emotional problems**	-0.71	-0.78	-0.38
	p < 0.001	p < 0.001	p=0.14
**Social functioning**	-0.81	-0.62	-0.60
	p < 0.001	p=0.014	p=0.015
**Mental health**	-0.64	-0.65	-0.46
	p=0.003	p=0.009	p=0.075
**Energy/Vitality**	-0.79	-0.70	-0.42
	p < 0.001	p=0.004	p=0.11
**Pain**	-0.66	-0.59	-0.60
	p=0.002	p=0.018	p=0.010
**General health perception**	-0.67	-0.68	-0.49
	p=0.002	p=0.006	p=0.056
**Change in health**	-0.58	-0.39	-0.63
	p=0.007	p=0.13	p=0.007
**Physical component score**	-0.59	-0.57	-0.55
	p=0.007	p=0.027	p=0.027
**Mental component score**	-0.88	-0.73	-0.45
	p < 0.001	p=0.002	p=0.082

If a Bonferroni correction were applied to account for the multiple domains of the SF-36, the p value is reduced to p < 0.0045, but this might represent an over correction.

## Discussion

This study shows that IIH compromises quality of life which significantly recovers after a therapeutic diet. Improving QOL in IIH is associated with resolving headache but there’s no evidence that it is associated with improvement in other clinical measures (vision, intracranial pressure and BMI). Patients and physicians may undervalue or may not be aware of the broad impact of IIH on health related quality of life. This study expands our knowledge of the quantitative adverse effects on patients and determines what factors can improve QOL in this disease. This study is in keeping with the current literature [8-10] that compared to the normal population QOL, as measured by the 36-Item Short Form Health Survey, QOL in IIH is reduced. This was most notable in the domains energy/vitality, pain and general health perception, p < 0.001.

There are characteristics, such as age, gender and obesity, which could potentially contribute to a lower QOL in IIH, a disease that mainly affects overweight women of child bearing age. For example females report poorer health, except in general health perception, p < 0.001 in UK normative data; there are age differences across the domain score in UK normative data, such as physical functioning being as high as 90.1 (16.4) in women aged 18–24, n=780 and as low as 84.8 (18.3), n=917 in women aged 45–54 [15]. Obesity is associated with a decrease in physical QOL in a dose–response relationship, while morbid obesity (Body Mass Index >40 kg/m^2^) has been shown to have significantly decreased mental QOL when assessed with 36-Item Short Form Health Survey compared to normal population results [9]. Surprisingly, our results demonstrate that significant weight loss was not associated with improved QOL. The lack of association between obesity and QOL in IIH has been demonstrated in a study which compared IIH to a healthy age-and weight- controls and found lower scores, suggesting that obesity alone cannot explain the lower QOL in IIH [8].

In this interventional study the measurable clinical parameters of vision, perimetry, papilloedema and intracranial pressure significantly improved; yet not one of these variables were significantly associated with enhanced QOL. We have, however, demonstrated a markedly significant relationship between positive QOL score and resolving headache. Our findings agree with earlier literature showing that headache severity correlates with quality of life, rather than headache frequency [16]. This potentially suggests that treatments to modifying headache severity are more important than altering headache frequency when treating IIH patients to improve QOL . Of additional interest was the more robust relationship between improving headache severity and QOL compared to improving headache frequency and QOL. Headache frequency is frequently regarded in the literature as the most important outcome measure in trials, yet our data suggests that in IIH it is headache severity which most affects quality of life. Of note, headache severity maybe more important that headache frequency in determining physical function, role limitation due to emotional problems, mental health, energy and vitality and general health perception and mental component score. Unsurprisingly, a reduction in analgesia use (days/week) correlated with an improvement in quality of life, particularly the pain domain. We evaluated, acetazolamide use, and found there to be no significant difference in QOL for those taking and not taking acetazolamide.

Health-related QOL outcomes are important in deciding whether an intervention is worth adopting [17,18]. A therapeutic weight loss intervention has already been shown to reduce intracranial hypertension, improve headaches, visual function and reduce papilloedema IIH [11]. Our study demonstrates that health-related QOL significant improves following a therapeutic diet, supporting its use as a suitable treatment for IIH.

There are a number of limitations to this study. Participant numbers included in this study were small, with 25 patients included at baseline and 20 patients completing all stages. This reduces our power to detect differences if they truly exist. We also accept that multiple comparisons were made due to the multiple components of the SF-36 score and clinical measures and accept that 5% of the results could have occurred by chance due to type 1 error. However, the vast majority of the correlations indicated a significant result with r values substantially different from zero which supports the results. Ideally, further studies are required looking at QOL longitudinally with weight loss interventions. Over the initial control stage, there was little significant change in the 36-Item Short Form Health Survey scores, except for energy/vitality and pain. These changes may reflect the positive psychological effect of entering into a research study [19]. Furthermore, some QOL domain measures may require extended periods of time before significant changes are observed. As this study was relatively short these potential differences may have been overlooked. Mental health, in particular, may require a longer period of observation to detect improvements compared to quicker resolving factors such as pain. AJS is currently developing the first national IIH database in the United Kingdom to enable longitudinal analysis. We did not evaluate co-morbid depression in our cohort but this would certain be of interest in future studies.

Modern studies are now reporting a lower incidence of blindness in IIH (1–3%), compared to previous studies 6–25% [1,6]. This may be due to increased recognition of the potentially blinding consequences of IIH in additional to improved management and monitoring of visual symptoms [1,6]. This is the first study to report the relationship between QOL and clinical outcomes.

## Conclusions

We have established that QOL significantly recovers after a therapeutic diet. Finally we have shown that improving QOL in IIH is associated with resolving headache and not with other clinical measures (vision, intracranial pressure and BMI). The management of IIH has typically focused on the visual parameters because of the risk of blindness, whilst active treatment of headache is frequently neglected [1,6]. This study highlights the importance of headache in determining QOL in IIH. We therefore recommend that specific headache treatment is key to improving QOL in IIH.

## Additional files

Additional file 1:
**Displays the mean changes in Short-Form Survey score over the observation period (stage 1) and therapeutic diet (stage 2).**
Additional file 2:
**Details the correlation between 36-Item Short Form Health Survey domains and clinical outcomes (visual function, ICP, papilloedema, BMI and weight).**
Additional file 3:
**Displays the correlation between changes in HIT-6 score (x axis) and change in individual 36-Item Short Form Health Survey domains (y axis) during therapeutic diet (stage 2).**

## Abbreviations

IIH: Idiopathic intracranial hypertension
QOL: Quality of life
SF-36: 36-item short form health survey
BMI: Body mass index
ICP: Intracranial pressure
HIT-6: Six-item headache impact test
LogMAR: Log of the minimum angle of resolution
OCT: Ocular coherence tomography
VAS: Visual analogue pain scale
