# Supplementary figures and images for: Headache determines quality of life in idiopathic intracranial hypertension
Source: J Headache Pain. 2015 May 15;16:45. doi: 10.1186/s10194-015-0521-9 (PMC4436432; doi:10.1186/s10194-015-0521-9)

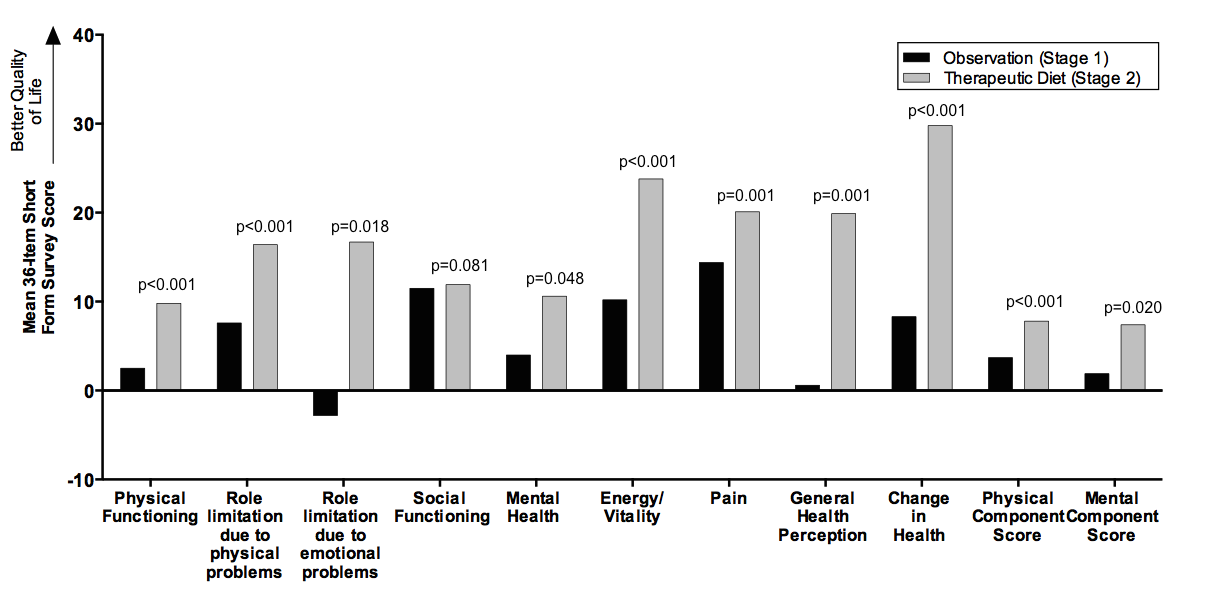

Supplement: Additional file 1: — Displays the mean changes in Short-Form Survey score over the observation period (stage 1) and therapeutic diet (stage 2). [file 10194_2015_521_MOESM1_ESM.tiff]

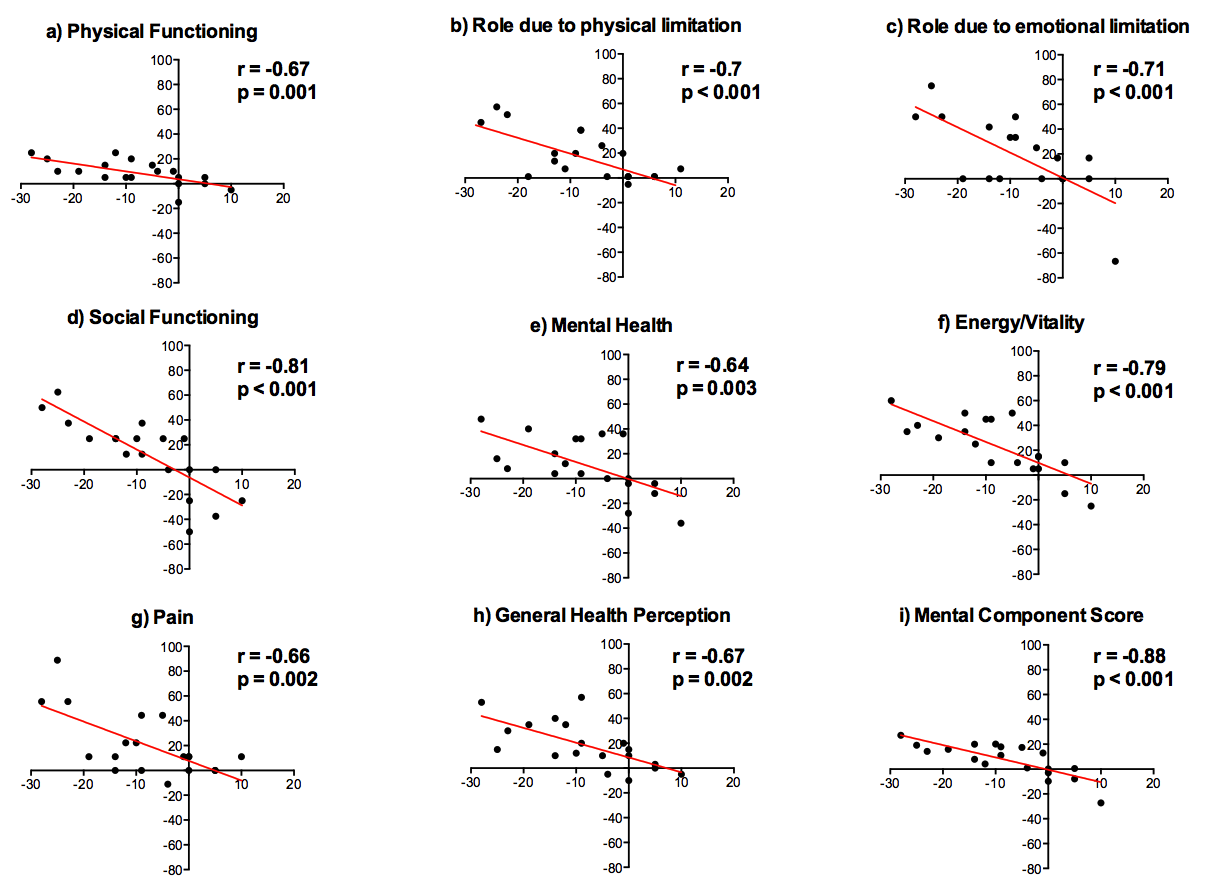

Supplement: Additional file 3: — Displays the correlation between changes in HIT-6 score (x axis) and change in individual 36-Item Short Form Health Survey domains (y axis) during therapeutic diet (stage 2). [file 10194_2015_521_MOESM3_ESM.tiff]
