# Supplementary material for: Headache determines quality of life in idiopathic intracranial hypertension
Source: J Headache Pain. 2015 May 15;16:45. doi: 10.1186/s10194-015-0521-9 (PMC4436432; doi:10.1186/s10194-015-0521-9)
Supplement: Additional file 2: — Details the correlation between 36-Item Short Form Health Survey domains and clinical outcomes (visual function, ICP, papilloedema, BMI and weight). [file 10194_2015_521_MOESM2_ESM.docx]

**Additional File 2**

Correlation between 36-Item Short Form Health Survey domains and clinical outcomes.

| **Domain** | **Humphrey’s Visual fields**  **(automated perimetry)** | [**Logarithm**](http://en.wikipedia.org/wiki/Logarithm) **of the Minimum Angle of Resolution visual acuity** | **ICP**  **(lumbar puncture)** | **Papilloedema (Optical Coherence Tomography)** | **Body Mass Index** | **Weight** |
| --- | --- | --- | --- | --- | --- | --- |
| **Physical Functioning** | 0.23  p=0.33 | 0.09  p=0.70 | -0.02  P=0.94 | -0.2  p=0.37 | -0.11  p=0.62 | -0.15  p= 0.51 |
| **Role limitation due to physical problems** | 0.10  p=0.68 | -0.15  p=0.53 | -0.30  p=0.91 | -0.37  p=0.097 | 0.34  p=0.13 | 0.34  p=0.13 |
| **Role limitation due to emotional problems** | 0.21  p=0.37 | 0.24  p=0.31 | 0.08  p=0.97 | -0.48  p=0.026 | 0.21  p=0.36 | 0.20  p=0.39 |
| **Social Functioning** | 0.03  p=0.91 | 0.12  p=0.61 | -0.09  p=0.69 | -0.38  p=0.097 | 0.10  p=0.66 | 0.04  p=0.86 |
| **Mental Health** | 0.19  p=0.44 | 0.23  p=0.34 | -.0.08  p=0.73 | -0.1  p=0.66 | -0.04  p=0.85 | -0.08  p=0.74 |
| **Energy/Vitality** | 0.21  p=0.39 | 0.35  p=0.14 | -0.12  p=0.61 | -0.10  p=0.67 | -0.26  p=0.27 | -0.26  p=0.27 |
| **Pain** | 0.08  p=0.73 | 0.25  p=0.29 | -0.34  p=0.13 | -0.49  p=0.024 | 0.05  p=0.82 | 0.05  p=0.82 |
| **General Health Perception** | 0.01  p=0.96 | 0.07  p=0.79 | -0.18  p=0.45 | 0.22  p=0.34 | -0.03  p=0.89 | -0.05  p=0.84 |
| **Change in Health** | -0.02  p=0.92 | 0.31  p=0.18 | -0.35  p=0.12 | -0.35  p=0.12 | -0.10  p=0.66 | -0.15  p=0.52 |
| **Physical Component Score** | 0.03  p=0.89 | -0.03  p=0.90 | -0.38  p=0.10 | -0.32  p=0.17 | 0.06  p=0.78 | 0.07  p=0.78 |
| **Mental Component Score** | 0.17  p=0.48 | 0.28  p=0.24 | -0.05  p=0.83 | -.0.25  p=0.28 | 0.01  p=0.96 | 0.02  p=0.92 |
